# The Role of lncRNAs in Rare Tumors with a Focus on HOX Transcript Antisense RNA (*HOTAIR*)

**DOI:** 10.3390/ijms221810160

**Published:** 2021-09-21

**Authors:** Giuseppina Liguori, Margherita Cerrone, Annarosaria De Chiara, Salvatore Tafuto, Maura Tracey de Bellis, Gerardo Botti, Maurizio Di Bonito, Monica Cantile

**Affiliations:** 1 Pathology Unit, Istituto Nazionale Tumori-Irccs-Fondazione G. Pascale, 80131 Naples, Italy; g.liguori@istitutotumori.na.it (G.L.); margherita.cerrone@istitutotumori.na.it (M.C.); a.dechiara@istitutotumori.na.it (A.D.C.); g.botti@istitutotumori.na.it (G.B.); m.dibonito@istitutotumori.na.it (M.D.B.); 2 Sarcomas and Rare Tumors Unit, Istituto Nazionale Tumori-Irccs-Fondazione G. Pascale, 80131 Naples, Italy; s.tafuto@istitutotumori.na.it; 3 Rehabilitation Medicine Unit, Istituto Nazionale Tumori-Irccs-Fondazione G. Pascale, 80131 Naples, Italy; maura.traceydebellis@istitutotumori.na.it

**Keywords:** lncRNAs, HOTAIR, rare cancers

## Abstract

Rare cancers are identified as those with an annual incidence of fewer than 6 per 100,000 persons and includes both epithelial and stromal tumors from different anatomical areas. The advancement of analytical methods has produced an accurate molecular characterization of most human cancers, suggesting a “molecular classification” that has allowed the establishment of increasingly personalized therapeutic strategies. However, the limited availability of rare cancer samples has resulted in very few therapeutic options for these tumors, often leading to poor prognosis. Long non coding RNAs (lncRNAs) are a class of non-coding RNAs mostly involved in tumor progression and drug response. In particular, the lncRNA HOX transcript antisense RNA (*HOTAIR*) represents an emergent diagnostic, prognostic and predictive biomarker in many human cancers. The aim of this review is to highlight the role of HOTAIR in rare cancers, proposing it as a new biomarker usable in the management of these tumors.

## 1. Introduction

Rare cancers are identified as diseases with an incidence of <15 cases per 100,000 people per year, as reported by the National Cancer Institute of the United States, or <6 per 100,000 people per year, as suggested by Surveillance of Rare Cancers in Europe (RARECARE) [1]. In Italy, the rare cancers group mainly includes rare epithelial tumors of the digestive system, followed by epithelial tumors of the head and neck, rare cancers of the female genital system, endocrine tumors, sarcomas, central nervous system tumors and rare epithelial tumors of the thoracic cavity [2].

Rare cancers account for approximately 20–25% of all cancer patients diagnosed each year, overall achieving a much higher incidence rate than any single common cancer. However, their low prevalence results in a lack of research funding, decreased awareness, late or misdiagnosis, few treatment options and limited clinical trials due to the small number of eligible patients.

Recently, new large-scale sequencing technologies have made it possible to molecularly characterize the majority of human cancers. However, most rare cancer studies have been limited to a small sample of patients [3,4].

All these factors result in poor prognosis for rare cancers causing a significant public health issue. Therefore, there is a need to establish large collections of rare tumor samples, preferably through the implementation of dedicated biobanks [5,6], and secondly, to define molecular characterization in order to identify new prognostic markers and therapeutic targets.

Only 2% of the human genome is transcribed and translated into proteins. About 70% of it is transcribed into ncRNA. LncRNAs represents a new class of RNA molecules, longer than 200 nucleotides, described as crucial biomarkers in cancer. They have secondary and three-dimensional structures which enable them to have both RNA- and protein-like functions [7]. It has been demonstrated that the majority of lncRNAs are localized in the nucleus [8] and in cytoplasm [9]. The change in cellular localization is associated with specific cellular functions in many cases. Nuclear lncRNA are mainly involved in chromatin regulation, transcription regulation and scaffolding, acting as platforms for the assembly of multiple-component complexes such as ribonucleoprotein (RNP) complexes. The role of cytoplasmic lncRNAs is mainly associated with post-transcriptional regulation, for example, sponging microRNAs; thereby, they reduce miRNA availability to target mRNA [10].

Recent studies have revealed that the deregulation of specific lncRNAs is widely involved in the development and progression of tumors, affecting molecular mechanisms associated with cell proliferation, migration, invasion, epithelial-to-mesenchymal transition (EMT) and apoptosis [11,12].

Between numerous cancer related lncRNAs, HOTAIR plays a main role in contributing to tumor development, metastatic progression and drug resistance. For its proven prognostic value, HOTAIR has also been suggested as a potential therapeutic target in human cancer [13].

Since little is known about the role of lncRNAs in low incidence cancers, and there is a need to identify new molecular markers and therapeutic targets for these neoplasms, in this review we will discuss the role of lncRNAs, with the focus on HOTAIR, in head and neck rare cancers, soft tissue tumors, neuroendocrine tumors, rare digestive system tumors and central nervous system tumors.

## 2. LncRNAs in Rare Tumors

An increasing number of ncRNAs, especially lncRNAs, were found to play crucial roles in the initiation and progression of rare cancers, suggesting that they could function as novel biomarkers and therapeutic targets [14,15] (Table 1).

Rare cancers of the head and neck are epithelial cancers of the larynx, hypopharynx, nasal cavity and sinuses, nasopharynx, major salivary glands and salivary-gland type tumors, oropharynx, oral cavity and lip, eye and adnexa and the middle ear. Apart from these tumors, other rare malignancies can be located in the head and neck region, such as soft tissue sarcoma, bone sarcoma and Merkel cell carcinoma [16].

The incidence of laryngeal carcinoma is relatively low, comprising between 2 and 5% of all malignant diseases diagnosed annually worldwide. More than 95% of laryngeal tumors are squamous cell carcinomas (LSCC) [16]. Different lncRNAs have been described as prognostic markers in this tumor [17]. Wu et al. [18] reported that the lncRNA H19 is necessary for the development and progression of LSCC. It is inversely correlated with the survival rate of LSCC patients, and its knockdown inhibits LSCC cells migration, invasion and proliferation. Moreover, H19 is able to promote LSCC progression via miR-148a-3p and DNA methyltransferase enzyme DNMT [18]. The lncRNA UCA1 (Urothelial Cancer Associated 1) expression in LSCC patients is significantly higher in tumor tissues compared with adjacent healthy tissues and its serum levels is increased in these patients compared to healthy controls. In vitro, UCA1 is able to promote cell proliferation, invasion and migration of LSCC cells by activating the Wnt/β-catenin signaling pathway [19]. Similarly, lncRNA small NF90-associated RNA (snaR) is upregulated in the plasma of patients with LSCC compared to healthy controls [20]. In LSCC patients with neck nodal metastasis, the lncRNA NEAT1 (nuclear paraspeckle assembly transcript1) is overexpressed and its gene silencing in vitro and in vivo models can inhibit tumor growth [21]. The overexpression of lncRNA PCAT19 (protocadherin 19) is strongly associated with decreased overall survival of LSCC patients and its silencing in cell lines decreases tumor growth in vivo by regulating the miR-182/PDK4 axis [22]. Many other lncRNAs are aberrantly expressed in LSCC tissues and are correlated with poor prognosis, such as LINC00668 [23], lncRNA TUG1 (taurine-upregulated gene 1) [24], HOXA11 antisense RNA (HOXA11-AS) [25], ATB [26], LINC02154 and MNX1 (motor neuron and pancreas homeobox 1)-AS1 [27].

**Table 1:** Main lncRNAs described in rare cancers.

LncRNAs	Tumor Type	Expression	References
*AOC4P*	GIST	upregulation	[28]
*ADAMTS9-AS2*	SACC	upregulation	[29]
*AFAP1-AS1*	NPC	upregulation	[30]
*ATB*	LSCC	upregulation	[26]
*BCAR4*	Osteosarcoma	upregulation	[31]
*CCDC26*	GIST	upregulation	[32,33]
*DNM3OS*	GIST	upregulation	[34]
*EWSAT1*	Ewing’s sarcoma	upregulation	[35]
*H19*	LSCC	upregulation	[18]
	NPC	upregulation	[36]
	NEN	upregulation	[37]
	GIST	upregulation	[28]
	Glioblastoma	upregulation	[38,39]
*HNF1A-AS*	NPC	upregulation	[36]
	Osteosarcoma	upregulation	[40]
*HOTTIP*	Osteosarcoma	upregulation	[41]
*HOXA11-AS*	LSCC	upregulation	[25]
	NPC	upregulation	[42,43]
*KCNQ1OT1*	NPC	upregulation	[44]
*LINC00668*	LSCC	upregulation	[23]
*LINC02154*	LSCC	upregulation	[27]
*MAGI2-AS3*	NPC	upregulation	[45]
*MALAT1*	Osteosarcoma	upregulation	[46]
	GEP-NEN	upregulation	[47]
	GIST	upregulation	[48]
	Glioblastoma	upregulation	[49,50,51,52,53]
*MINCR*	NPC	upregulation	[54]
*MRPL23-AS1*	SACC	upregulation	[55]
*NEAT1*	LSCC	upregulation	[21]
	Glioblastoma	upregulation	[56]
*NONHSAT154433.1*	MEC	upregulation	[57]
*OIP5-AS1*	NPC	upregulation	[58]
*PCA3*	Pulmonary NEN	upregulation	[59]
*PCAT19*	LSCC	upregulation	[22]
*PILRLS*	Liposarcoma	upregulation	[60]
*SNHG8*	NPC	upregulation	[61]
*SNHG15*	NPC	upregulation	[62]
*TP73-AS1*	NPC	upregulation	[63]
*TUG1*	LSCC	upregulation	[24]
	Osteosarcoma	upregulation	[64]
*TUSC7*	Glioblastoma	upregulation	[65]
*UCA1*	LSCC	upregulation	[19]
	Osteosarcoma	upregulation	[66]
*LET*	NPC	downregulation	[67]
*MEG3*	Osteosarcoma	downregulation	[68]
	Pulmonary NEN	downregulation	[59]
*TUS7*	Osteosarcoma	downregulation	[68,69]

Nasopharyngeal carcinoma (NPC) is a rare tumor arising from the epithelial cells that cover the surface and line the nasopharynx, with an annual incidence rate of 1/100,000 in Western countries. Although rare, NPC accounts for about one third of childhood nasopharyngeal tumors [16].

The lncRNA actin filament-associated protein 1 antisense RNA1 (AFAP1-AS1) is upregulated in NPC and associated with poor survival. Its silencing significantly inhibited NPC cells migration and invasion [30]. In the same manner, the lncRNA H19 and hepatocyte nuclear factor 1A-antisense RNA (HNF1A-AS) are overexpressed in NPC tissues and involved in the modulation of cell cycle progression, tumor cell proliferation, migration and epithelial to mesenchymal transition (EMT) [36,70]. On the contrary, the downregulation of lncRNA-low expression in tumor (lncRNA-LET) in NPC tissues is significantly correlated to advanced clinical stage, tumor size, lymph node metastases and poor survival of patients [67].

More recently, many other lncRNAs have been associated with NPC progression, with their capacity of sponging different microRNAs [58,61,62,63,71,72,73,74]. Additionally, in NPC patients, a large number of lncRNAs were also associated with cisplatin resistance [42,43,44,45,75,76,77,78] and radioresistance [54,79,80].

Salivary gland cancer (SGC) comprises a heterogeneous group of tumors with approximately 6.5% of the cases among the malignant tumors of the head and neck. They are considered rare cancers, having an annual incidence of less than 2/100,000 in most countries [81]. There are very few indications of the role of lncRNAs in salivary gland tumors.

An lncRNA microarray analysis highlighted that lncRNA ADAM metallopeptidase with thrombospondin type 1 motif, 9 (ADAMTS9) antisense RNA 2 (ADAMTS9-AS2) was significantly upregulated in salivary adenoid cystic carcinoma (SACC) and it is correlated with metastasis rate and poor prognosis in SACC patients [29]. Similarly, the long non-coding RNA (lncRNA) MRPL23 antisense RNA 1 (MRPL23-AS1) was highly expressed and correlated with lung metastasis and overall survival rate in patients with SACC [55]. More recently, the upregulation of lncRNA NONHSAT154433.1 and decreased expression of circ012342 have been closely related to the pathogenesis of mucoepidermoid carcinoma (MEC) [57].

Soft tissue sarcomas are relatively uncommon tumors, accounting for 1% of all malignancies. They are classified in about 80 histologic subtypes according to tissue components from which they are derived. Approximately 80% of sarcomas originate from soft tissues, while 20% from bone [82].

Many lncRNAs are involved in osteosarcoma (OS) progression. The lncRNA MALAT1 (metastasis-associated lung adenocarcinoma transcript 1) was closely correlated with lung metastasis in OS patients, and it is an independent prognostic factor of OS. Its knockdown affected the PI3K/AT signaling pathway and inhibited invasion and metastasis in vitro and in vivo [46]. In OS tissues, other lncRNAs are significantly upregulated and related with metastatic progression, such as HNF1A (HNF1 homeobox A)-AS1 [40], BCAR4 (breast cancer anti-estrogen resistance 4) [31] and HULC (highly upregulated in liver cancer RNA) [83]. The latter was strongly correlated with a shorter overall survival in OS patients [83]. In a same manner, HOTTIP (HOXA distal transcript antisense RNA) [41], UCA1 [66] and TUG1 [64] expression increased in osteosarcoma tissue and was associated with poorer overall survival. On the contrary, MEG3 (maternally expressed 3) and TUS7 are downregulated in human osteosarcoma tissue [68,69]. Emerging studies suggest that lncRNAs contribute to tumor cell growth and proliferation in Ewing’s sarcoma. The lncRNA EWS-AT1 (EWS RNA binding protein 1) was found to be induced and upregulated by EWS-FLI1 chimeric protein in primary pediatric human mesenchymal progenitor cells. EWSAT1 diminished cell viability in human Ewing sarcoma cell lines [35]. LncRNA PILRLS (Proliferation Interacting LncRNA in Retroperitoneal Liposarcoma) was overexpressed in retroperitoneal liposarcoma, and its silencing is able to significantly inhibit cell proliferation and colony formation of liposarcoma cells [60].

Neuroendocrine neoplasms (NENs) are a relatively rare and heterogeneous tumor types, accounting for about 0.5% of all newly diagnosed malignancies [84]. The most frequent primary sites are the gastrointestinal tract (62–67%) the lungs (22–27%), and more rarely the genitourinary tract [85].

Very little is known about the role of lncRNAs in epigenetic regulation of NENs development and progression. Expression levels of lncRNAs MALAT1 and HOTAIR analyzed by chromogenic in situ hybridization (ISH) were associated with tumor stages and development of metastases in GEP-NEN [47]. H19 was significantly upregulated in NEN tissues with malignant behaviors, and its upregulation is able to predict poor prognosis. In vitro and in vivo data showed that H19 overexpression promoted tumor growth and metastasis and revealed that H19 activated PI3K/AKT/CREB signaling and promoted pNEN progression by interacting with VGF (*VGF* nerve growth factor inducible) [37]. LncRNA-p21 is highly expressed in neuroendocrine prostate cancer patients and cells [86] while MEG3 and prostate cancer antigen 3 (PCA3) were aberrantly expressed in pulmonary NENs, including typical carcinoid tumors, atypical carcinoid tumors, small cell lung carcinoma (SCLC/NEC) and large cell neuroendocrine carcinoma (LCNEC/NEC) [59].

Digestive system tumors form in organs involved in digesting food and drinks and some of them belong to the category of rare tumors, such as cholangiocarcinoma and gastrointestinal stromal tumors (GISTs) [87]. Badalamenti et al. [48] analyzed the expression of H19 and MALAT1 in 40 metastatic GIST tissues, showing their upregulation in 50% of GIST patients. Both H19 and MALAT1 overexpression was significantly higher in patients with time to progression (TTP) < 6 months as compared to patients with TTP > 6 months. Moreover, MALAT1 expression levels seem to be correlated with c-KIT mutation status [48]. The aberrant expression of lncRNA AOC4P has been detected in high-risk GISTs compared with low- and intermediate-risk GISTs. In addition, its expression appeared closely associated with upregulation of epithelial–mesenchymal transition (EMT)-related proteins, such as TGF-β (*transforming growth factor*-*beta*), ZEB1 (Zinc finger E-Box binding homeobox 1), Vimentin, Snail, and E-cadherin. AOC4P silencing led to the decrease in cell proliferative migration and invasive ability of GIST cells [28]. Next-generation sequencing data of paired GIST and adjacent tissue samples were analyzed by a web-based lincRNA analysis, showing the deregulation of lncRNAs MALAT1, H19 and FENDRR (FOXF1 adjacent noncoding developmental regulatory RNA). Moreover, H19 upregulation appeared strongly related with different oncogenes, such as ETV1 (ETS variant transcription factor 1) and miR-455-3p [88]. The deregulation of other lncRNAs has been recently described in GIST. Cao et al. [32] described the role of the lncRNA coiled-coil domain-containing 26 (CCDC26) in imatinib resistance of GIST, highlighting that cells with lower CCDC26 expression were less sensitive to imatinib compared to those with higher CCDC26 expression. In addition, CCDC26 expression decreased in a time-dependent manner in the presence of imatinib and its silencing can upregulate c-KIT expression [32]. Recently, an oncomine analysis performed on a large series of low-risk and high-risk GISTs, revealed that the lncRNA DNM3OS was involved in the malignant transformation of GISTs and correlated with a worse prognosis. Finally, DNM3OS was involved in the Hippo signaling pathway by regulating the expression of GLUT4 (glucose transporter member 4) and CD36 [34].

Central nervous system (CNS) tumors are relatively rare, and they are associated with high morbidity and mortality. The most common glial tumors are glioblastoma multiform and anaplastic glioma, comprising more than 50 and 10%, respectively, of the total gliomas [89]. Abnormal expression of several lncRNAs have been detected in glioma/glioblastoma tumors and related cell lines. In particular, MALAT1 has been noted to be involved in the pathogenesis of glioblastoma. Vassallo et al. revealed that MALAT1 silencing is able to decrease glioblastoma cells migration, without affecting proliferation [49]. Most studies report the role of MALAT 1 as an important marker of chemoresistance to TMZ (temozolomide). It can enhance the resistance of glioma cells to TMZ by regulating ZEB1 [50]. Different molecular pathways are associated with resistance mechanism related to MALAT 1 in glioblastoma cells. Chen et al. reported that MALAT1 induces chemoresistance to TMZ through suppressing miR-203 expression and promoting the expression of thymidylate synthase [51]. Similarly, Cai et al. reported the upregulation of MALAT1 and its main role in TMZ-resistant glioblastoma cells by inhibiting the miR-101 signaling pathway [52]. More recently, NF-κB and p53 have been identified as regulators of the MALAT1 expression in induction of TMZ resistance in glioblastoma [53]. The serum levels of MALAT1 have also been associated with poor response to TMZ and lower survival rate of patients with glioblastoma [51]. H19 is another oncogenic lncRNA in glioblastoma whose aberrant expression is inversely correlated with the expression of NKD1 (NKD inhibitor of WNT signaling pathway 1), an inhibitor of the Wnt pathway [38]. Similar to MALAT1, H19 silencing is also able to modulate TMZ cytotoxicity in glioma cells by inhibiting EMT via the Wnt/β-catenin pathway and inactivating NF-κB signaling [39]. Other lncRNAs have been described as involved in TMZ resistance in glioblastoma cells, such as NEAT1 [56] and TUSC7 (tumor suppressor candidate 7) [65].

## 3. HOTAIR and Its Role in Human Cancers

HOTAIR is a lncRNA located within the intergenic region between HOXC11 and HOXC12 in the HOXC cluster on chromosome 12q13.13. Its principal transcript is 2364 bp RNA, transcribed from a 6449 bp gene locus and composed of six exons [90]. The human HOTAIR gene can be transcribed into several variants via alternative splicing and recently six major HOTAIR splicing variants have been described [91]. HOTAIR promoter contains binding sites for numerous transcription factors, which include AP1, Sp1, ERE elements, HRE elements and NF-κB [13]. HOTAIR, as well as many lncRNAs, is a key modulator of chromatin stability and is mainly involved in transcriptional silencing mechanisms [91]. Mechanistically, HOTAIR is able to bind the *PRC2* (Polycomb repressive complex) at the 5′ end, and the *LSD1* (lysine-specific histone demethylase 1A) at the 3′ end, acting as a molecular scaffold for the conjunction of the two complexes [90,91,92]. The HOTAIR-PRC2-LSD1 complex determines epigenetic changes contributing to targeted gene silencing and repressing their transcription via H3K27 trimethylation (PRC2 activity) and H3K4 demethylation (LSD1 activity) [91,92]. HOTAIR can also modulate gene expression at the post-transcriptional level describing that it could serve as a ubiquitination protein and subsequent degradation platform [93].

Physiologically, HOTAIR can be involved in the regulation of the cell cycle. It promotes the cell cycle that passes through the restriction point during the G1 phase by regulating CDK4/6-cyclin D and the Rb-E2F pathway [94]. During embryogenesis, *HOTAIR* is involved in the development of the lumbosacral region, through the repression of HOX D locus genes [90]. Furthermore, it was reported that HOTAIR possesses many miRNA recognition elements (MREs), and their functional interactions are able to modulate important cellular processes [95,96,97].

Numerous studies have shown that HOTAIR can be directly associated with tumor diseases being involved in tumor initiation, growth, angiogenesis, progression, recurrence and drug resistance mechanisms [92,98,99]. In addition, many clinical studies suggested HOTAIR as a fundamental biomarker associated with poor prognosis [100]. Early studies highlighted that the aberrant expression of *HOTAIR* in human tumors have been detected in breast cancer (BC) patients. *HOTAIR* appears to be a powerful predictor of BC tumor progression: its upregulation has been described in primary BC tumors with high metastatic potential and poor survival [101]. The deregulation of HOTAIR expression has been found in different molecular subtypes of BC often with conflicting data [102,103,104,105]. In BC, HOTAIR is also involved in the regulation of many different processes, mainly related with epithelial mesenchymal transition (EMT) [106]. BC cells are able to promote the EMT and metastasis processes, when treated with TGF-B1, through the upregulation of HOTAIR. The downregulation of HOTAIR results in the reduction of the ability to form colonies [106,107]. Similarly, the promotion of metastatic processes in BC is strongly influenced by the interaction of HOTAIR with a series of microRNAs. In BC, HOTAIR is able to interact with different miRNAs promoting tumor progression, such as miR-7 [108], miR-206 [95] and miR34a [96,109]. HOTAIR has been detected in the blood of BC patients, and its circulating DNA level significantly correlated with the clinical stage of the tumor [110]. Moreover, Tang et al. [111] showed that serum exosomal *HOTAIR* is a potent predictor of both poor survival and drug response in BC patients [111]. Different studies showed the crucial prognostic role of HOTAIR also in gastrointestinal tract tumors, especially in colorectal cancer [112] and gastric cancer [113]. In the latter, high level of circulating HOTAIR is associated with sensibility to fluorouracil and platinum-based combination therapy [113]. In liver cancer, *HOTAIR* upregulation correlates with clinical-pathological features and tumor progression [114] and its silencing increases chemotherapy sensitivity [115]. In urogenital cancers, the role of *HOTAIR* has been well documented, especially in prostate [116] and bladder cancer [117,118,119]. *HOTAIR* is overexpressed in ovarian cancer, and it is associated with stage, lymph node metastases and poor survival [120]; similarly, HOTAIR is overexpressed in cervical cancer [121,122] and in endometrial carcinoma [123] in which it is also associated with cisplatin resistance acquisition [124]. Finally, *HOTAIR* upregulation correlates with advanced stage, lymph nodes metastases, poor prognosis and drug resistance in non-small cell lung cancer (NSCLC) patients [125,126]

## 4. The Role of HOTAIR in Rare Tumors

**Figure 1:**
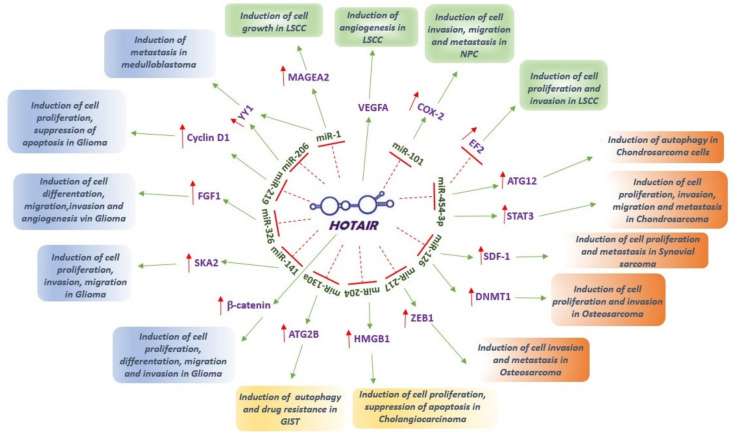
Schematic representation of the main molecular pathways related to HOTAIR deregulation in rare cancers. In rare tumors of the head and neck rare (green box), HOTAIR promotes cell growth, proliferation, migration and invasion through the direct interaction and consequent inhibition of different miRNAs: HOTAIR/miR-1 functional interaction promotes MAGEA2 expression, which is able to suppress p53-dependent apoptosis in response to drugs, decrease cellular senescence and increase cell proliferation in LSCC cells; HOTAIR inhibits miR-454-3p that target EF2, the principal target of the tumor suppressor pRB, reducing tumor suppression in LSCC cells; HOTAIR/miR-101 functional interaction in NPC cells induces COX2 expression, a regulator of tumor metabolism, angiogenesis and tumor microenvironment. In LSCC cells, HOTAIR interacts with VEGFA promoting angiogenic processes. In soft tissue tumors (orange box), HOTAIR interacts with different miRNAs leading to their inhibition and promoting cell proliferation, migration and invasion of tumor cells: HOTAIR/miR-454-3p functional interaction induces (i) ATG12 expression, a positive regulator of autophagic vesicle formation, and (ii) STAT3, involved in regulation of cancer inflammation and metastasis, in chondrosarcoma cells. HOTAIR/miR-126 functional interaction promotes (i) SDF-1 expression, the main receptor of the metastasis inducer gene CXCR4 able to modulate proliferation/survival and EMT of synovial sarcoma cells, and (ii) DNMT1, involved in cancer stem cell maintenance and tumorigenesis, in osteosarcoma cells. HOTAIR inhibits miR-217 upregulating ZEB1, a potent inducer of EMT, in osteosarcoma cells. In rare digestive system tumors (yellow box), HOTAIR interacts with miR-204 to upregulate HMGB1 gene, a crucial regulator of inflammation and cancer progression, in cholangiosarcoma cells, and with miR-130a to induce ATG2B expression promoting autophagy in GIST cells. In central nervous system tumors (blue box), HOTAIR is able to induce b-catenin expression, modulating Wnt signal pathways in glioma cells, and establishes functional interaction with different miRNAs to promote cell proliferation invasion and migration: HOTAIR/miR-141 interaction induce upregulation of SKA2, a gene involved in cell cycle regulation, in glioma cells; HOTAIR/miR-326 interaction leads to the upregulation of FGF1, involved in the repairing progress of damaged, in glioma cells; HOTAIR inhibits miR-219 upregulating Cyclin D1, a key regulator of cell cycle progression, in glioma cells; HOTAIR/miR206 functional interaction induces YY1 expression promoting metastatic progression in medulloblastoma cells. Green arrow connects related pathways and function; Red arrow indicates induction of expression; T indicates inhibition of expression.

A series of studies carried out with in vitro and in vivo models of rare cancers highlighted the fundamental role that HOTAIR has in these tumors and the complex network of molecular interactions in which it is involved. Its aberrant activity is capable of regulating the main molecular pathways associated with carcinogenesis, metastatic progression, angiogenesis and drug resistance (Figure 1). Furthermore, most of these studies highlighted the direct correlation between HOTAIR upregulation and the prognosis of patients with rare tumors. In Table 2, the main groups of rare tumors are summarized on the basis of their incidence (carcinomas and sarcomas).

### 4.1. Head and Neck Cancers

The aberrant expression of lncRNA HOTAIR has been abundantly described in head and neck tumors, especially in rare epithelial cancers such as laryngeal squamous cell carcinoma, nasopharyngeal carcinoma and salivary gland tumors.

HOTAIR was upregulated in primary LSCC, compared with adjacent noncancerous tissues and its overexpression was correlated with poor differentiation, lymph node metastasis and advanced clinical stages. HOTAIR silencing in LSCC cells leads to a significant decrease in invasive ability and promotes apoptosis. Furthermore, HOTAIR knockdown can effectively suppress the progression of LSCC in vivo xenografts mice [139]. The combined expression of HOTAIR and its interactor, which is an enhancer of zeste homolog 2 (*EZH2*, a regulator of epigenetic modification), were overexpressed in LSCC tissue. HOTAIR overexpression is significantly related to T phase, pathological grading and metastatic risk. Its silencing promoted cell proliferation and increased sensitivity to cis-platinum in the LSCC cells [127]. Moreover, a large case series of LSCC has been selected to evaluate the influences of cisplatin and paclitaxel on lncRNAs expression. HOTAIR was dramatically reduced with the increasing concentration of cisplatin and paclitaxel suggesting their target function on specific lncRNASs in LSCC patients [128]. Recently, a bioinformatics analysis to examine miRNAs, lncRNAs and mRNAs differentially expressed was performed on recurrent and non-recurrent LSCC sample datasets. Analysis showed that HOTAIR, HCG4 (HLA complex group 4) and EMX2OS (EMX2 opposite strand/antisense RNA) can represent a non-coding RNA signature in recurrent LSCC. Furthermore, the HOTAIR-miR-1-MAGEA2 (melanoma antigen A2 gene) interaction may be fundamental for the identification of recurrent LSCC [140].

HOTAIR’s role as a circulating marker has been extensively documented in the majority of solid cancers and a series of studies validated this role in rare tumors. A large study conducted on 52 LSCC patients and 49 patients with benign polyps of the vocal cords showed that the expression of exosomal HOTAIR was significantly higher only in patients with LSCC. In addition, patients with lymph node metastasis had higher serum exosomal HOTAIR expressions than those with no metastases, suggesting that circulating HOTAIR could be a valuable biomarker to screen and predict progression for LSCC patient [141]. Finally, exosome-mediated HOTAIR is able to act as ceRNA of miR-454-3p to regulate the tumor suppressor gene E2F2 (Eukaryotic elongation factor 2), negatively regulating the radiosensitivity of laryngeal cancer cells [142].

In nasopharyngeal carcinoma patients, HOTAIR expression levels increased with clinical stage progression, and it is associated with poor prognosis. The functional analysis in in vitro models showed that *HOTAIR* is able to modulate migration, invasion and proliferation of NPC cells [129]. Subsequent studies have validated the prognostic role of HOTAIR in NPC and highlighted a strong relationship with angiogenic pathways. Functional studies exhibited that silencing of HOTAIR by siHotair directly inactivated VEGFA transcriptional activity and suppressed the expression of glucose regulated protein 78 (GRP78); this suggests its main role in mediating tumorigenesis and angiogenesis in NPC [130]. Some other molecular pathways are related with aberrant expression of HOTAIR in NPC patients. The expression of fatty acid synthase (FASN) is positively correlated to HOTAIR and de novo synthesis of cellular free fatty acid in NPC cells is inhibited when HOTAIR was silenced [143]. HOTAIR is able to induce COX-2 (Cyclooxygenase-2) upregulation and promotes proliferation, migration and invasion in NPC cells. Moreover, miR-101 directly binds to the 3’-UTR of COX-2 and downregulates COX-2 expression, suggesting the importance of HOTAIR/miR-101/COX-2 axis in progression of nasopharyngeal carcinoma cells [144]. More recently, Yang et al. demonstrated that HOTAIR inhibits E-cadherin by stimulating the trimethylation of H3K27 to promote NPC cell progression through recruiting histone methylase EZH2 [145].

There is some evidence of the role of HOTAIR in salivary gland cancers. A recent study conducted on 86 patients with salivary adenoid cystic carcinoma (SACC) showed that HOTAIR expression in SACC tissue was higher than that in normal salivary gland tissue. Additionally, its expression in tissues of patients with TNM stages III or IV, nerve invasion, lymph node metastasis and poor survival rate is increased, suggesting that HOTAIR is a potential marker for prognostic assessment of patients with SACC [131].

### 4.2. Neuroendocrine Tumors

Numerous lncRNAs have been associated with neuroendocrine tumors pathogenesis especially in gastroenteropancreatic neuroendocrine tumors (GEP-NET); however, the role of HOTAIR is still poorly investigated.

Upregulation of HOTAIR has been described in GEP-NET and it is significantly associated with grade and aggressive phenotype [47]. More recently, this data has been confirmed. In fact, HOTAIR showed weak expression in low-grade GEP NENs and aberrant expression in NET G3 and NEC G3 categories. Furthermore, HOTAIR appeared inversely correlated with posterior HOX genes expression, highlighting that the combined expression can be useful in molecular stratification of GEP-NENs [146].

Regarding prostate cancer, Chang et al. [136] described that HOTAIR is upregulated in castration-resistant PCa (CRPC) with neuroendocrine differentiation (NEPC). Specifically, HOTAIR overexpression is sufficient to induce NED, whereas knockdown of HOTAIR suppressed it in PCa cells. In fact, HOTAIR upregulation induced the expression of some NEPC markers in prostate cancer cells. HOTAIR expression can be inhibited by the transcriptional repressor REST (RE1 silencing transcription factor), which is a master transcriptional repressor that restricts neuronal gene expression in stem cells and non-neuronal cell [136]. However, HOTAIR’s role in this process has recently been downsized. Mather et al. [147] found that, while REST is consistently downregulated in NEPC versus CRPC/adenocarcinoma samples, *HOTAIR* is expressed at very similar levels in the two groups, suggesting that the protein REST plays a pivotal role in inhibiting NEPC transdifferentiation, and that this effect is not mediated by *HOTAIR* [147].

Finally, a recent study analyzed HOTAIR expression in typical carcinoid tumors, atypical carcinoid tumors, small cell lung carcinoma (SCLC/NEC) and large cell neuroendocrine carcinoma (LCNEC/NEC), highlighting its upregulation only in SCLC/NEC patients [59].

### 4.3. Rare Digestive System Tumors

Among rare digestive system tumors, cholangiocarcinoma represents a heterogeneous group of highly aggressive malignancies originating from the biliary ducts with poor prognosis [148].

HOTAIR was highly expressed both in cholangiocarcinoma tissues and cell lines compared with corresponding normal bile duct tissues and intrahepatic biliary epithelial cells. Its overexpression is strongly correlated with tumor size, TNM stage and recurrence in cholangiocarcinoma patients. HOTAIR silencing significantly decreased the migration and invasion and increased apoptosis of cholangiocarcinoma cell models [149]. More recently, Lu et al. demonstrated that HOTAIR is able to promote cholangiocarcinoma progression by regulating HMGB1 to suppress cell apoptosis, autophagy and induce cell proliferation by sponging miR-204-5p [150]. The analysis of polymorphisms in the gene sequence of HOTAIR to evaluate the susceptibility to the development of cholangiocarcinoma has recently been evaluated. In a Greek cohort of patients, HOTAIR rs4759314 AG and GG genotypes were associated with a significantly increased cholangiocarcinoma risk [137].

GISTs are rare, making up less than 1% of all gastrointestinal tumors. HOTAIR appeared overexpressed in GISTs and the combined overexpression of miR-196a are strongly associated with high-risk grade, metastasis and poorer patient survival. Knockdown of HOTAIR altered the expression of GIST repressed cells invasiveness [138]. Lee et al. [151] described the upregulation of HOTAIR in surgically resected high-risk GISTs compared with low- and intermediate-risk GISTs. In GIST cell models, HOTAIR is able to repress apoptosis and to promote cell invasion and migration. Furthermore, HOTAIR induces methylation of PCDH10 (Protocadherin 10), a *tumor* suppressor *gene*, in GIST cells [151]. Several other studies confirmed the upregulation of HOTAIR in GISTs with a high risk of recurrence. Bure et al. highlighted that HOTAIR knockdown in GIST cells modulates the expression of genes involved in the organization and disassembly of the extracellular matrix and induces locus-specific alterations of DNA methylation patterns, especially in DPP4 (dipeptidyl peptidase 4), RASSF1 (Ras association domain family member 1) and ALDH1A3 (aldehyde dehydrogenase 1 family member A3) genes [152]. Furthermore, HOTAIR is described as a drug resistance-related lncRNA in GIST which is involved in imatinib resistance [153]. More recently, Zhang et al. [132] analyzed HOTAIR expression in GIST cells after imatinib treatments showed that HOTAIR is able to shift from nucleus to cytoplasm thereby modulating drug sensitivity via autophagy. In addition, HOTAIR, downregulating miRNA-130a and thereby activating the downstream target autophagy-related protein 2 homolog B (ATG2B), is able to modulate autophagy and imatinib sensitivity in GIST cells [132].

### 4.4. Central Nervous System Tumors

HOTAIR aberrant expression has been abundantly described in glioma tumors and closely associated with glioma grade and poor prognosis [154]. HOTAIR expression correlated with glioma molecular subtype and was preferentially expressed in the classical and mesenchymal subtypes compared with the neural and proneural subtypes. HOTAIR silencing induced colony formation suppression, cell cycle G0/G1 arrest and orthotopic tumor growth inhibition, acting as a crucial regulator of cell cycle progression in glioma cells [154]. The same authors described that EZH2 inhibition blocked cell cycle progression in glioma cells, consistent with the effects elicited by HOTAIR siRNA, suggesting that HOTAIR might regulate cell cycle progression through EZH2 [155]. Several molecular pathways have been regulated by HOTAIR in glioma. NLK (Nemo-like kinase), a negative regulator of the β-catenin pathway, was negatively correlated with HOTAIR expression. When the β-catenin pathway was inhibited, glioma cells became susceptible to cell cycle arrest and inhibition of invasion. HOTAIR expression induction in in vivo model of glioma upregulated β-catenin, while its silencing inhibited glioma cell migration/invasion [156]. Bromodomain and extraterminal (BET) proteins are important therapeutic targets in glioblastoma. Treatment of glioblastoma cells with the BET bromodomain inhibitor I-BET151 reduced levels of HOTAIR and restored the expression of several other glioblastoma downregulated lncRNAs. Moreover, bromodomain containing 4 (BRD4) is able to bind to HOTAIR, directly regulating its expression [133].

Different miRNAs can interact with HOTAIR during glioma progression. HOTAIR is the target of miR-326 and its silencing promotes their tumor-suppressive effects on glioma cell lines. Moreover, overexpressed miR-326 reduced the FGF1 expression, which played an oncogenic role in glioma by activating PI3K/AKT and MEK 1/2 pathways [157]. Bian et al. [158] showed that HOTAIR might act as an endogenous ‘sponge’ of miR-141, thereby regulating the de-repression of SKA2 (spindle and kinetochore associated complex subunit 2), a gene involved in cell cycle regulation. Both overexpression of miR-141 and knockdown of HOTAIR in a mouse model of human glioma resulted in significant reduction of tumor growth in vivo [158]. More recently, the interaction of HOTAIR and miR-219 has been described in glioma cells. HOTAIR silencing strongly induced the expression of miR-219 reducing cell proliferation and promoting apoptosis. Concomitantly, the protein expression level of Cyclin D1 declined significantly suggesting that HOTAIR can repress the proliferation and promote the apoptosis of glioblastoma cells by targeting miR-219 [33]. Circulating levels of HOTAIR have also been described in glioma patients and are associated with a poor prognosis [159]. Tan et al. detected high HOTAIR levels in serum samples from GBM patients and the serum-derived exosomes containing HOTAIR were significantly correlated with high grade brain tumors [134]. Regarding the analysis of polymorphisms in the HOTAIR sequence related with glioma susceptibility, Xavier-Magalhães et al. [160] reported a case-control study consisting of 177 Portuguese glioma patients and 199 cancer-free controls. HOTAIR SNPs rs920778 and rs12826786 do not play a significant role in glioma susceptibility but may be important prognostic factors in anaplastic oligodendroglioma [160].

Transcriptome analysis was carried out to evaluate the expression of the HOX genes and HOTAIR in several pediatric tumors such as teratoid rhabdoid tumors (ATRT), ependymomas, medulloblastomas, glioblastoma multiforme and juvenile pilocytic astrocytomas (JPAs). HOTAIR appeared overexpressed in ATRTs, medulloblastomas and JPAs, and downregulated in ependymomas [161]. Nevertheless, a more recent study analyzed HOTAIR expression in adult myxopapillary ependymoma (MPE), highlighting its overexpression compared with non-ependymoma spinal tumors [162].

HOTAIR upregulation has also been detected in medulloblastoma tissues and cell lines. In medulloblastoma cells, HOTAIR is able to negatively regulate miR-1 and miR-206 expression which can directly target YY1, a transcription factor described as a metastasis inducer. Finally, HOTAIR knockdown suppressed medulloblastoma cell proliferation, tumor growth, migration and invasion, and promoted cell apoptosis via the modulation of the miR-1/miR-206-YY1 axis, as well as EMT [163].

### 4.5. Soft Tissue Tumors

The role of HOTAIR in soft tissue sarcomas is mainly related to specific tumor types. Milhem et al. [164] selected primary and metastatic tumor samples from myxofibrosarcoma, synovial sarcoma, leiomyosarcoma and malignant fibrous histiocytoma sarcoma subtypes to analyze HOTAIR expression. In these tumor types, high levels of HOTAIR are correlated with a high probability of metastatic progression. In contrast, reduced expression of HOTAIR is correlated with a good response to treatment in terms of necrosis, suggesting that HOTAIR can be considered a useful predictor for metastatic risk and outcome of therapeutic treatments [164]. Many studies are focused on osteosarcoma (OS), highlighting the main role of HOTAIR as prognostic biomarker. HOTAIR is highly expressed in OS tumor samples and cells and its upregulation was closely correlated with advanced tumor stage along with highly histological grade. Furthermore, a high level of HOTAIR was significantly associated with shorter overall survival [165]. HOTAIR silencing leads to the downregulation of DNA methyltransferase 1 (DNMT1), promoting the decrease in global DNA methylation level. HOTAIR is able to induce the expression of DNMT1 through repressing miR-126, which is the negative regulator of DNMT1. Furthermore, HOTAIR silencing increases the sensibility of OS cells to DNMT1 inhibitor through regulating the viability and apoptosis of OS cells via HOTAIR-miR126-DNMT1-CDKN2A axis [135]. Wang et al. [166], using bioinformatics analysis, showed that HOTAIR can be targeted by the tumor suppressive gene miR-217. In addition, HOTAIR siRNA increased miR-217 expression and significantly repressed osteosarcoma cell growth, migration, invasion and induced cell apoptosis capacity. ZEB1 was identified as a downstream gene of miR-217, suggesting that HOTAIR can mediate osteosarcoma progression by upregulating ZEB1 expression via acting as a competitive endogenous RNA (ceRNA) via miR-217 [166]. Recently Wang et al. [167] described that LPS (a major component of Gram-negative bacteria) promotes tumor invasion, metastasis and EMT in osteosarcoma. HOTAIR expression markedly increases in LPS-induced EMT in osteosarcoma cells, such as TLR4 (Toll Like Receptor 4), which is the LPS receptor, suggesting that the effects of LPS on EMT in osteosarcoma cells is mediated via the TLR4/HOTAIR pathway [167]. Several studies reported that different polymorphisms, especially in intronic sequences as well as in promoter regions of HOTAIR, are often associated with its aberrant expression, patient prognosis and cancer susceptibility in different tumor types [99]. Zhou et al. [168] identified a SNP located in HOTAIR gene (rs7958904) that was significantly associated with decreased risk of OS. Furthermore, subjects with the rs7958904 CC genotype had significantly lower HOTAIR RNA levels than other genotypes [168].

Sporadic information on the role of HOTAIR has been associated with other types of sarcomas. In chondrosarcoma patients, the expression of HOTAIR is correlated with tumor stage and poor prognosis. HOTAIR knockdown led to growth inhibition via G0/G1 arrest and apoptosis in vitro and in vivo models of chondrosarcoma [169]. HOTAIR expression was increased in chondrosarcoma tissues compared with normal cartilage tissues and its aberrant expression was elevated in high-grade compared with low-grade chondrosarcoma tissues. In addition, overall survival time of patients with high expression of HOTAIR was significantly shorter than that of patients with low expression of HOTAIR [169]. HOTAIR is able to induce DNA methylation of miR-454-3p by recruiting EZH2 and DNMT1 in chondrosarcoma cells. Furthermore, signal transducer and activator of transcription 3 (STAT3) and autophagy-related gene 12 (*ATG12*) are targets of miR-454-3p, initiate HOTAIR deficiency-induced apoptosis and reduce autophagy [169]. More recently, Feng et al. [138] described the aberrant expression of HOTAIR also in synovial sarcoma (SS). Overexpression of HOTAIR correlates with histological grade, AJCC staging and distant metastasis [138]. In SS cells, HOTAIR silencing inhibited cellular proliferation, invasion and migration, promoting the G_1_/G_0_ phase of the cell cycle, and inhibiting the G_2_/S phase. Finally, HOTAIR knockdown increased miR-126 expression level and decreased the expression level of stromal cell-derived factor-1 (SDF-1) [170].

## 5. Conclusions

The understanding of the emerging role of lncRNAs in the main cell processes related with cancer development and progression represents a significant advance in oncology. LncRNAs are implicated in numerous biological processes such as cell cycle control, apoptosis, differentiation and epigenetic regulation of gene expression, becoming valid diagnostic and prognostic markers of human cancers [171,172]. Furthermore, the lncRNAs appear to be optimal biomarkers which are more stable in body fluids (urine, blood, saliva) and can be detected by using simple in situ (ISH) and molecular techniques [173]. The crucial role of lncRNAs, and in particular HOTAIR, is well documented in common human cancer such as breast, lung, urogenital and gastrointestinal cancer [172]. To date, the molecular characterization of the rarest cancers in the population has provided little information on potential new biomarkers and therapeutic molecular targets. However, much experimental evidence highlights the crucial role that HOTAIR plays in these tumors. The implementation of targeted functional studies could help to better understand how to interfere/block the aberrant activity of this biomarker, providing a new tool for the management of rare tumors. More recently, the design of small molecules able to specifically interfere with conserved RNA structures and to block HOTAIR protein complexes have proved more useful [174,175]. The direct or indirect block/inhibition of HOTAIR may represent a new and effective therapeutic strategy for rare cancer and tumors.

## Figures and Tables

**Table 2:** Principal roles of HOTAIR in patients with rare tumors.

	Tumor Types	Clinical Studies	References
Head and Neck rare cancers	LSCC	HOTAIR overexpression correlates with poor differentiation, pathological grade, metastatic risk and chemotherapy sensibility. HOTAIR is a circulating markers in LSCC patients.	[127,128]
	NPC	HOTAIR overexpression correlates with clinical stage and poor prognosis.	[129,130]
	SACC	HOTAIR overexpression correlates with clinical stage, nerve invasion, metastatic risk and poor survival.	[131]
Neuroendocrine tumors	GEP NEN	HOTAIR overexpression correlates with tumor grade and tumor stage.	[47]
Rare digestive system tumors	Cholangiocarcinoma	HOTAIR overexpression correlates with tumor size, TNM stage and metastatic risk.	[132]
	GIST	HOTAIR overexpression correlates with tumor grade, recurrence and metastatic risk, poor survival and drug resistance.	[133]
Central Nervous System tumors	Glioma/Glioblastoma	HOTAIR overexpression correlates with tumor grade, molecular subtype and poor prognosis. HOTAIR is a circulating markers in glioma patients.	[134,135]
Soft tissue tumors	Osteosarcoma	HOTAIR overexpression correlates with advanced tumor stage, high histological grade and shorter overall survival.	[136]
	Chondrosarcoma	HOTAIR overexpression correlates with tumor grade and shorter overall survival.	[137]
	Synovial sarcoma	HOTAIR overexpression correlates with histological grade, advanced tumor stage and metastatic risk.	[138]

## Abbreviations

HOTAIR	HOX transcript antisense RNA
EMT	Epithelial-to-mesenchymal transition
LSCC	Laryngeal tumors are squamous cell carcinomas
DNMT	DNA methyltransferase enzyme
UCA1	Urothelial Cancer Associated 1
NEAT1	Nuclear paraspeckle assembly transcript1
PCAT19	Protocadherin 19
TUG1	Taurine-upregulated gene 1
HOXA11-AS	HOXA11 antisense RNA
MNX1	Motor Neuron And Pancreas Homeobox 1)-AS1
NPC	Nasopharyngeal carcinoma
AFAP1-AS1	Actin filament-associated protein 1 antisense RNA1 ()
LET	Low Expression in Tumor
SGC	Salivary gland cancer
ADAMTS9AS2	ADAM metallopeptidase thrombospondin type 1, 9 antisense RNA 2
SACC	Salivary adenoid cystic carcinoma
MRPL23-AS1	MRPL23 antisense RNA 1
MEC	Mucoepidermoid carcinoma
OS	osteosarcoma
MALAT1	Metastasis Associated Lung Adenocarcinoma Transcript 1
HNF1A	HNF1 Homeobox A-AS1
BCAR4	Breast Cancer Anti-Estrogen Resistance 4
HULC	Highly upregulated in liver cancer RNA
HOTTIP	HOXA Distal Transcript Antisense RNA
MEG3	Maternally expressed 3
EWS-AT1	EWS RNA Binding Protein 1
PILRLS	Proliferation Interacting LncRNA in Retroperitoneal Liposarcoma
NEN	Neuroendocrine neoplasm
ISH	In situ hybridization
VGF	*VGF* Nerve Growth Factor Inducible
PCA3	Prostate cancer antigen 3
GISTs	Gastrointestinal stromal tumors
TGF-β	*Transforming growth factor-beta*
ZEB1	Zinc Finger E-Box Binding Homeobox 1
FENDRR	FOXF1 Adjacent Non-Coding Developmental Regulatory RNA
CCDC26	Coiled-coil domain-containing 26
GLUT4	Glucose transporter member 4
TMZ	Temozolomide
NKD1	NKD Inhibitor of WNT Signaling Pathway 1
TUSC7	Tumor Suppressor Candidate 7
*PRC2*	Polycomb repressive complex
*LSD1*	Lysine-specific histone demethylase 1A
MRE	miRNA recognition element
*EZH2*	Zeste homolog 2
HCG4	HLA Complex Group 4
EMX2OS	EMX2 Opposite Strand/Antisense RNA
MAGEA2	Melanoma Antigen A2 gene
E2F2	Eukaryotic elongation factor 2
GRP78	Glucose regulated protein 78
FASN	Fatty acid synthase
COX-2	Cyclooxygenase-2
TLR4	Toll Like Receptor 4
STAT3	Signal transducer and activator of transcription 3
*ATG12*	Autophagy-related gene 12
SDF-1	Stromal cell-derived factor-1
GEP-NET	Gastroenteropancreatic Neuroendocrine Tumors
REST	RE1 Silencing Transcription Factor
PCDH10	Protocadherin 10
DPP4	Dipeptidyl peptidase 4
RASSF1	Ras Association Domain Family Member 1
ALDH1A3	Aldehyde Dehydrogenase 1 Family Member A3
ATG2B	Autophagy-related protein 2 homolog B
BRD4	Bromodomain Containing 4
SKA2	Spindle And Kinetochore Associated Complex Subunit 2
ATRT	Teratoid rhabdoid tumors
MPE	Myxopapillary ependymoma
